# MicroRNA-1908-5p contributes to the oncogenic function of the splicing factor SRSF3

**DOI:** 10.18632/oncotarget.14184

**Published:** 2016-12-26

**Authors:** Hye Ree Kim, Chang Hoon Shin, Hong Lee, Kyung Hee Choi, Do-Hyun Nam, Takbum Ohn, Hyeon Ho Kim

**Affiliations:** ^1^ Department of Health Sciences and Technology, Samsung Advanced Institute for Health Sciences and Technology, Sungkyunkwan University, Seoul, Republic of Korea; ^2^ Department of Neurosurgery, Samsung Medical Center, Sungkyunkwan University School of Medicine, Seoul, Republic of Korea; ^3^ Department of Cellular and Molecular Medicine, College of Medicine, Chosun University, Gwangju, Republic of Korea; ^4^ Research Institute for Future Medicine, Samsung Medical Center, Seoul, Republic of Korea

**Keywords:** SRSF3, FADS1, miR-1908-5p, NF-κB, NKIRAS2

## Abstract

Serine/arginine (SR)-rich proteins that contain RS domains and SR repeats have diverse cellular functions including transcription, polyadenylation, translation, and RNA export. The splicing factor SRSF3, also termed SRp20, is the smallest member of the SR protein family and is a known proto-oncogene. Although it is implicated in the malignant phenotypes of various cancer cells, the molecular mechanism underlying SRSF3-mediated cancer progression is still obscure. We investigated here the oncogenic functions of SRSF3 in osteosarcoma U2OS cells. Knockdown of SRSF3 inhibited proliferation, clonogenicity, and metastatic potential including migration and invasion. It also decreased the level of miR-1908 independent of its host gene *FADS1*. Although *FADS1* was not associated with SRSF3-mediated malignant properties, overexpression of miR-1908-5p increased cell proliferation, migration, and invasion, suggesting that miR-1908-5p is responsible for the oncogenic functions of SRSF3. Knockdown of SRSF3 decreased the expression of miR-1908-5p by inhibiting transactivation of NF-κB. We observed that miR-1908-5p downregulated NF-κB inhibitor interacting Ras-like 2 (*NKIRAS2*), a negative regulator of the NF-κB pathway by directly binding to the 3’UTR of *NKIRAS2* mRNA. Consistent with overexpression of miR-1908-5p, knockdown of NKIRAS2 diminished the expression level of IκB-β and provoked translocation of NF-κB into the nucleus where it transcriptionally activates its target genes including miR-1908-5p expression, thus elevating the proliferation and metastatic potential. Taken together, our results demonstrate that SRSF3 confers the malignant characteristics on cancer cells via the SRSF3/miR-1908-5p/NKIRAS2 axis.

## INTRODUCTION

Serine/arginine (SR)-rich proteins containing at least one RNA recognition motif (RRM) and one arginine/serine repeat (RS domain) have diverse cellular functions including transcription, polyadenylation, translation, and RNA export [1]. After SF2/ASF was identified as the first SR protein, others were subsequently classified on the basis of their molecular weight: SRp20, SRp40, SRp55, and SRp75 [2]. Analyses based on cross-linking and immunoprecipitation coupled with high-throughput sequencing (iCLIP-seq) determine that SR proteins are able to bind to functionally diverse mRNAs including non-coding RNAs, suggesting that disruption of their functions may cause various diseases [3]. As a critical regulator of RNA metabolism, SR proteins have received attention from oncologists. Several reports have revealed that SR proteins play critical roles in cancer progression [4].

SRSF3 (SR-rich splicing factor 3, also termed SRp20) is the smallest member of the SR protein family and is known proto-oncogene [4, 5]. However, the oncogenic roles of SRSF3 remain largely unknown. SRSF3 is frequently upregulated in most types of cancer and is closely associated with the prognosis of cancer patients. In various systems, SRSF3 confers malignant phenotypes including increased proliferation, anti-apoptosis, and gain of metastatic abilities (migration and invasion) [6–8]. SRSF3 has been recently reported to regulate alternative splicing and gene expression of forkhead box M1 (FoxM1), polo-like kinase 1 (PLK1), and cell division cycle 25B (Cdc25B) in U2OS osteosarcoma cells [4]. SRSF3 also translationally suppresses the expression of programmed cell death 4 (PDCD4) mRNA through an interaction with 5’ untranslated region (UTR) of its mRNA [9].

MicroRNAs (miRNAs) are an example of many regulatory non-coding RNAs and are 19~25 nucleotides in length. They play an important role in the regulation of gene expression through translational suppression or mRNA degradation mainly by targeting 3’ UTR of target mRNA [10]. Depending on their target genes, miRNAs are subdivided into oncogenic and tumor-suppressing miRNAs which suppress the expression of tumor suppressors and oncogenes, respectively. Accordingly, miRNAs widely influence cancer development and progression such as proliferation, anti-apoptosis, resistance, migration, and invasion [11]. For most miRNAs, primary miRNAs (pri-miRNAs) are transcribed by RNA polymerase II, either as separated or embedded transcripts. Although few miRNAs are known to have their own transcription factors, the majority of miRNAs are transcribed by transcription factors for their host genes.

In this study, we investigated the oncogenic function of SRSF3 in osteosarcoma U2OS cells. Our data reveals that SRSF3 confers malignant phenotypes such as increased proliferation, migration, and invasion on U2OS cells by upregulating NF-κB-dependent oncogenic miR-1908-5p expression. We also found that the NF-κB inhibitor interacting Ras-like 2 (*NKIRAS2*) gene is a novel target of miR-1908-5p and is responsible for the reinforcement of the NF-κB pathway by SRSF3/miR-1908-5p/NKIRAS2.

## RESULTS

### Knockdown of SRSF3 inhibits clonogenicity and metastatic potential of U2OS cells

**Figure 1:**
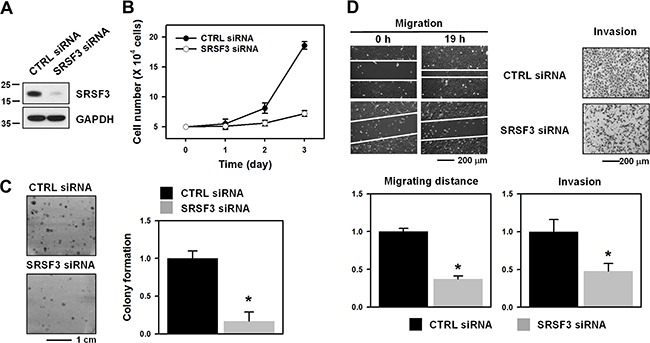
SRSF3 contributes to the malignant properties of U2OS cells **A**. U2OS cells were transfected with control (CTRL) or SRSF3-specific siRNA. The expression level of SRSF3 was determined by western blot analysis and GAPDH was used as a loading control. **B**. The equal number of transfected cells was resuspended into 12-well plates and cellular proliferation was assessed by counting the number of viable cells at every 24 h. **C**. For the clonogenic assay, transfected cells were plated into 6-well plates and cultured for more than 2 weeks. Clonogenic activity was assessed by counting the number of colonies. **D**. The migratory and invasive abilities were assessed by a wound healing assay and Matrigel invasion assay, respectively, as described in Materials and Methods. All experiments are performed more than three times and data represent the mean ± S.D. Asterisk (*) indicates statistical significance of p<0.05 as determined by Student's t-test.

Growing evidences demonstrate that several SR proteins including SRSF1 [12], SRSF3 [4], and SRSF6 [13, 14] are classified into oncogenes that are involved in rapid proliferation and metastatic properties. We investigated the effect of SRSF3 silencing on clonogenicity and metastatic potential of osteosarcoma U2OS cells. Short interference RNA (siRNA) was used to specifically knockdown SRSF3 expression. siRNAs targeting the coding region or 3’UTR of *SRSF3* mRNA efficiently decreased the expression of SRSF3 (Figure 1A and Supplementary Figure 1A). SRSF3-knockdown U2OS cells exhibited reduced proliferation as compared to the control siRNA-transfected cells (Figure 1B). We also found that reduced expression of SRSF3 inhibited clonogenicity (Figure 1C and Supplementary Figure 1B) and suppressed metastatic abilities including invasion (Figure 1D, left panel and Supplementary Figure 1C) and migration (Figure 1D, right panel and Supplementary Figure 1D), indicating that SRSF3 is responsible for the malignant phenotypes of osteosarcoma U2OS cells.

### SRSF3-mediated regulation of miR-1908 is independent of its host gene FADS1

**Figure 2:**
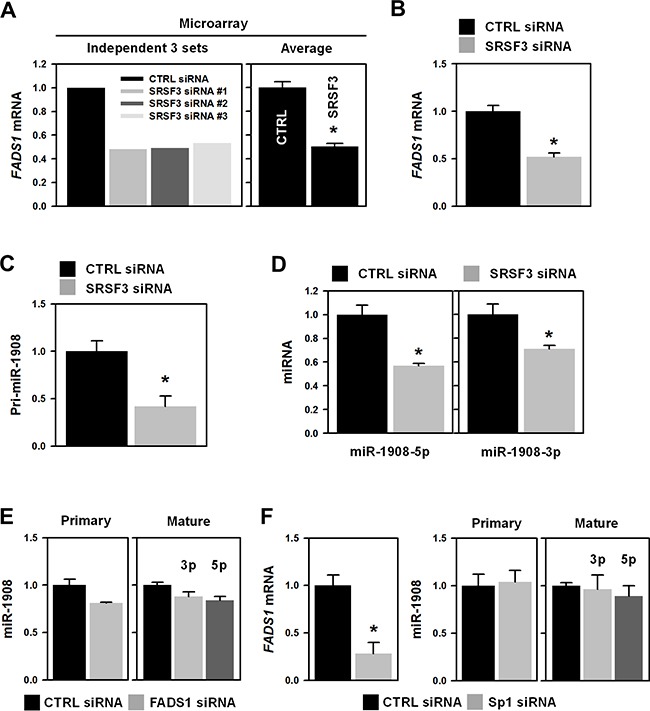
Knockdown of SRSF3 decreased the expression of miR-1908 and its host gene, FADS1 **A**. Microarray data showing decreased expression of FADS1 in SRSF3-silenced U2OS cells with three different siRNAs. **B**. U2OS cells were transfected with control (CTRL) or SRSF3-specific siRNA for 48 h and then the expression level of FADS1 mRNA was determined by RT-qPCR analysis. **C-D**. The levels of pri-miR-1908 and mature miR-1908-3p/5p were assessed by RT-qPCR analysis using pri-miR-1908-specific primers and TaqMan miRNA primers, respectively. **E**. Cells were transfected with control (CTRL) or FADS1-specific siRNA for 48 h and then the levels of pri-miR-1908 and mature miR-1908-3p/5p were assessed by RT-qPCR as described above. **F**. Cells were transfected with control (CTRL) or Sp1-specific siRNA for 48 h. The expression level of FADS1 mRNA was determined by RT-qPCR analysis and the levels of pri-miR-1908 and mature miR-1908 (3p/5p) were assessed by RT-qPCR analysis as described above. All experiments are performed more than three times and data represent the mean ± S.D. Asterisk (*) indicates statistical significance of p<0.05 as determined by Student's t-test.

A list of differentially expressed genes by the knockdown of SRSF3 was previously reported [9]. Based on this, we hypothesized that SRSF3 is able to control the expression of miRNAs and screened SRSF3-targeted genes harboring primary sequences of miRNAs in their introns. Among the 444 downregulated and 579 upregulated genes, only 40 genes (21 downregulated and 19 upregulated genes) harbor the primary sequences of miRNA (Supplementary Figure 2). Fatty acid desaturase 1 (*FADS1*) is one of the SRSF3 target genes and that contains the primary sequence of miR-1908 (pri-miR-1908) in its intron 1. By analyzing the microarray data, we found that *FADS1* mRNA was decreased in SRSF3-silenced U2OS cells (Figure 2A). For validation, U2OS cells were transfected with SRSF3-specific siRNA and the level of *FADS1* mRNA was assessed by RT-qPCR. Knockdown of SRSF3 significantly decreased the level of *FADS1* mRNA (Figure 2B and Supplementary Figure 3A). It also decreased the level of pri-miR-1908 (Figure 2C and Supplementary Figure 3B) and miR-1908-3p and miR-1908-5p, two strands of its mature form as well (Figure 2D and Supplementary Figure 3C). These results indicated that SRSF3 is able to regulate the expression of miR-1908 and its host gene *FADS1*. To check whether *FADS1* is involved in the decreased expression of miR-1908 by the SRSF3 knockdown, we tested the level of miR-1908 in *FADS1*-silenced cells (Figure 2E). It was observed that the expression of miR-1908 was not affected by FADS1. Most intronic miRNAs are usually regulated by transcription factor of their host genes and as previously found, SRSF3 regulates the expression of various transcription factors such as Sp1, which is predicted as a putative transcriptional factor of *FADS1* [15]. Given the marked decrease of Sp1 in SRSF3-silenced cells, we tested whether Sp1 is involved in the SRSF3-mediated regulation of miR-1908. Interestingly, we found that knockdown of Sp1 did not affect the expression of miR-1908 although *FADS1* mRNA was decreased in Sp1-silenced cells (Figure 2F and Supplementary Figure 3D). These results revealed that SRSF3 regulates the expression of miR-1908 independently from its host gene *FADS1*, suggesting that miR-1908 could be affected by its own transcription factors.

### *FADS1* is not involved in the oncogenic function of SRSF3

Since the knockdown of SRSF3 was found to decrease the expression level of *FADS1*, we investigated whether downregulation of *FADS1* is required for the inhibitory effects of SRSF3 silencing. Designed *FADS1*-specific siRNA effectively suppressed the expression of FADS1 (Supplementary Figure 4A). Unexpectedly, knockdown of *FADS1* did not influence proliferation and clonogenicity of U2OS cells (Supplementary Figure 4B and 4C, respectively). Furthermore, the metastatic potential such as invasive and migratory abilities was not affected in *FADS1*-silenced cells (Supplementary Figure 4D and 4E, respectively). These results indicated that *FADS1* is not associated with the oncogenic function of SRSF3.

### miR-1908-5p confers malignant properties on U2OS cells

**Figure 3:**
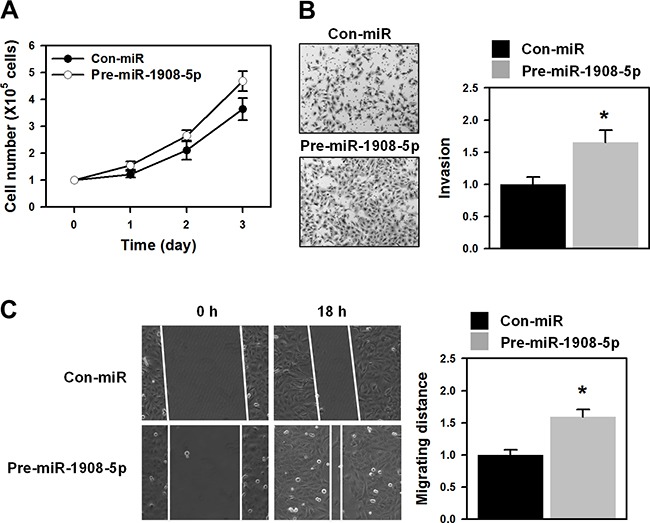
miR-1908-5p increased proliferation and metastatic potential **A**. U2OS cells were transfected with control (Con-miR) or pre-miR-1908-5p for 24 h and then resuspended into 12-well plates. The proliferation rate was determined by counting the number of viable cells at every 24 h. **B-C**. The invasive (B) and migratory (C) activities of miR-1908-5p-overexpressed cells were assessed by a wound healing assay and Matrigel invasion assay, respectively. All experiments are performed more than three times and data represent the mean ± S.D. Asterisk (*) indicates statistical significance of p<0.05 as determined by Student's t-test.

Previously, we found that SRSF3 contributes to the malignant properties of U2OS cells and *FADS1*, one of its target genes, is not responsible for the oncogenic function of SRSF3. Therefore, we checked whether miR-1908 is required for SRSF3-mediated malignancy. Although SRSF3 regulated the expression of both mature miRNAs, we further studied the roles of miR-1908-5p because its expression was more affected by the SRSF3 knockdown than its sister miRNA, miR-1908-3p. To examine the effects of miR-1908-5p on cellular proliferation, U2OS cells were transfected with *SRSF3*-specific siRNA and the proliferation rate was determined by counting viable cells every 24 h. U2OS cells highly expressing miR-1908-5p showed an increased proliferation rate compared to parental cells (Figure 3A). In addition to proliferation, we also checked the effect of miR-1908-5p on metastatic properties. The invasive and migratory abilities were strengthened by overexpression of miR-1908-5p (Figure 3B and 3C). From the above results, we demonstrated that miR-1908-5p is closely associated with the oncogenic functions of SRSF3.

### The NF-κB pathway is implicated in the upregulation of miR-1908 by SRSF3

**Figure 4:**
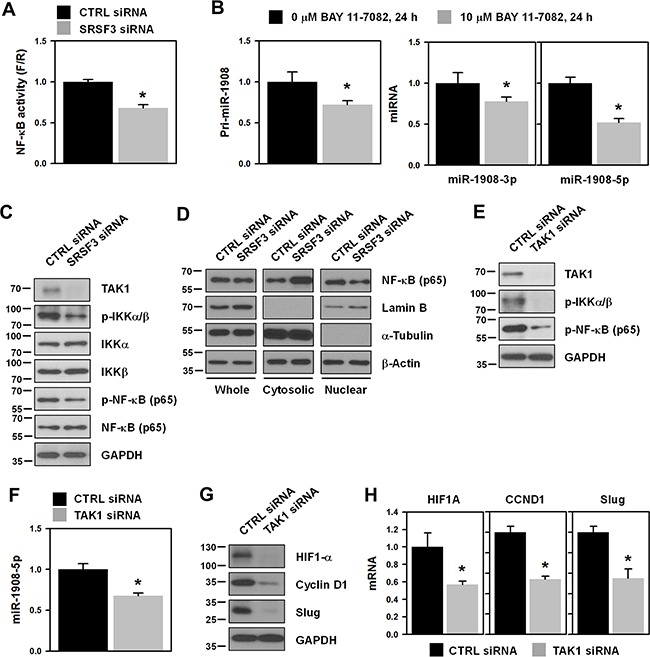
NF-κB is involved in SRSF3-regulated miR-1908 expression **A**. U2OS cells were transfected with control (CTRL) or SRSF3-specific siRNA. Transfected cells were resuspended into 12-well plates and then transfected with the NF-κB reporter vector. Transactivation of NF-κB was assessed by calculating the luciferase activity. **B**. To check whether NF-κB regulates the expression of miR-1908, cells were treated with the NF-κB-specific inhibitor BAY11-7082 (10 μM) for 24 h. The level of miR-1908-3p/5p was determined by RT-qPCR analysis using TaqMan primers. **C**. Cells were transfected with control (CTRL) or SRSF3-specific siRNA for 48 h and then whole cell lysates were prepared. Western blot analyses were performed using adequate specific antibodies. **D**. Cells were transfected as described above and then cellular fractionation was performed. Western blot analysis was performed to determine the localization of NF-κB (p65) and α-tubulin and lamin B was checked for cytoplasmic and nuclear markers, respectively. **E-F**. Cells were transfected with control (CTRL) or TAK1-specific siRNA and then the levels of TAK1 and phosphorylated IKKα/β and NF-κB were assessed by Western blot analysis. The level of miR-1908-5p was determined by RT-qPCR analysis. **G-H**. The protein and mRNA expression levels of NF-κB target genes including HIF1-α, cyclin D1, and slug, were determined by western blot and RT-qPCR analysis, respectively. All experiments are performed more than three times and data represent the mean ± S.D. Asterisk (*) indicates statistical significance of p<0.05 as determined by Student's t-test.

We realized that SRSF3-mediated regulation of miR-1908 is unconnected with the expression of its host gene *FADS1*; therefore, we sought the molecular mechanism underlying how SRSF3 regulates miR-1908 expression. NF-κB has recently been identified as a transcriptional factor of miR-1908 [16]. Bioinformatics analysis revealed two putative NF-κB binding sites in the 1243 bp miR-1908 promoter located in the intron of *FADS1* [16]. With this in mind, we tested whether SFSR3 can activate the NF-κB pathway. First, we checked the effect of SRSF3 silencing on the transcriptional activity of NF-κB using a reporter vector (Figure 4A). Transactivation of NF-κB was diminished in *SRSF3*-silenced cells. Secondary, we tested whether NF-κB is implicated in SRSF3-regulated miR-1908 expression. U2OS cells were treated with the NF-κB inhibitor BAY 11-7082 for 24 h and then the levels of pri-miR-1908 and miR-1908-3p/5p were assessed by RT-qPCR. As shown in Figure 4B, the expressions of primary (left panel) and mature form (right panel) of miR-1908 were decreased by the inhibition of NF-κB, revealing that SRSF3 regulates the expression of miR-1908 through the NF-κB pathway. By investigating the molecular mechanism underlying the roles of SRSF3 in the NF-κB pathway, we found that SRSF3 is able to affect NF-κB transactivation through transforming growth factor-β-activated kinase 1 (TAK1). TAK1 is known to regulate NF-κB transactivation through IKKα/β-mediated phosphorylation of NF-κB (p65) [17]. Knockdown of SRSF3 decreased the expression of TAK1 and thus suppressed the phosphorylation of IKKα/β and NF-κB (p65 subunit), indicating that SRSF3 is responsible for the activation of the NF-κB pathway (Figure 4C). To verify this SRSF3-dependent activation, we checked the cytoplasmic and nuclear localization of NF-κB in SRSF3-silenced cells (Figure 4D). Knockdown of SRSF3 increased the level of cytoplasmic localization; however, the level of nuclear NF-κB (p65) was decreased in *SRSF3*-silenced cells, indicating that SRSF3 activates the NF-κB pathway by triggering the nuclear localization of NF-κB (p65). To test whether TAK1 is responsible for SRSF3-mediated NF-κB activation, U2OS cells were transfected with control (CTRL) or *TAK1*-specific siRNA for 48 h and then the phosphorylated levels of IKKα/β and NF-κB (p65) were assessed (Figure 4E). Knockdown of TAK1 inhibited the phosphorylation of IKKα/β and NF-κB. These results indicate that SRSF3 is able to positively influence the NF-κB pathway by regulating the expression of TAK1. We also checked the level of miR-1908-5p and NF-κB target genes in *TAK1*-silenced cells. Knockdown of TAK1 decreased the expression level of miR-1908-5p (Figure 4F). It also showed decreased protein and mRNA levels of NF-κB target genes (Figures 4G and 4H, respectively).

### miR-1908-5p suppresses NKIRAS2 expression by directly binding to the 3’UTR of its mRNA

**Figure 5:**
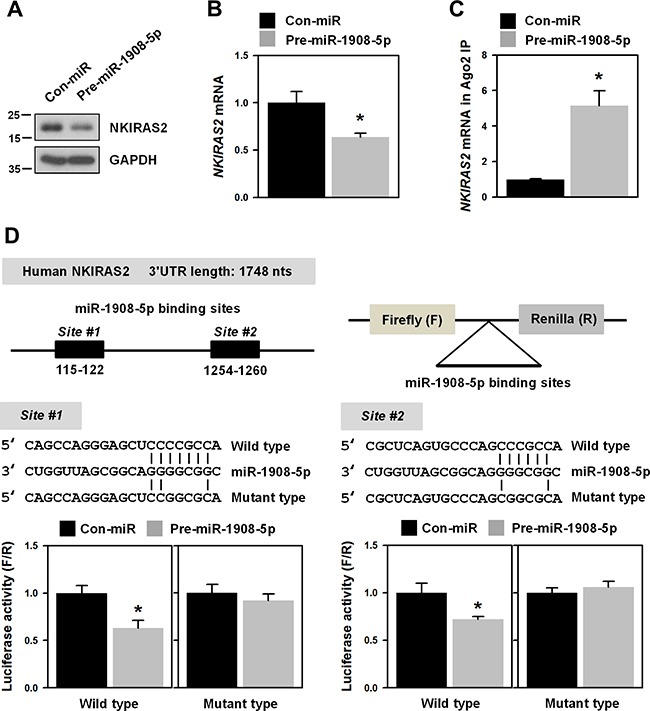
NKIRAS2 is a novel target of miR-1908-5p **A-B**. U2OS cells were transfected with control (Con-miR) or pre-miR-1908-5p for 48 h and then the level of NKIRAS2 protein and mRNA was assessed by western blot and RT-qPCR analysis, respectively. **C**. To check direct interaction between miRNA and target mRNA, Ago2 immunoprecipitation (IP) was performed using an Ago2-specifc antibody. Cytoplasmic lysates were prepared from control (CTRL) or pre-miR-1908-5p-transfectected cells and used for Ago2-IP. The mRNAs bound to miRNA-Ago2 were isolated and the level of NKIRAS2 mRNA was assessed by RT-qPCR analysis. **D**. To verify miR-1908-5p suppressed NKIRAS2, luciferase vectors harboring wild-type and mutated miR-1908-5p-binding sequences were manufactured. Luciferase activity was assessed as described in Materials and Methods. All experiments are performed more than three times and data represent the mean ± S.D. Asterisk (*) indicates statistical significance of p<0.05 as determined by Student's t-test.

By screening miR-1908-5p target genes, we paid attention to *NKIRAS2*, a negative regulator of the NF-κB pathway. First, we checked the effect of miR-1908-5p on the expression of *NKIRAS2* in U2OS cells. Overexpression of miR-1908-5p decreased the expression of NKIRAS2 and *NKIRAS2* mRNA (Figures 5A and 5B, respectively). To test whether miR-1908-5p directly binds to *NKIRAS2* mRNA, Ago2 immunoprecipitation (IP) was performed using an Ago2-specific antibody (Figure 5C). Compared to the control, *NKIRAS2* mRNA was more enriched in Ago2 IP materials due to overexpression of miR-1908-5p, indicating that the miR-1908-5p-loaded RISC complex interacts with *NKIRAS2* mRNA. To verify the interaction between miR-1908-5p and *NKIRAS2* mRNA, luciferase vectors containing wild-type and mutated sequences for miR-1908-5p were manufactured (Figure 5D). Since two binding sites are predicted, we manufactured two different reporter vectors. In both vectors, overexpression of miR-1908-5p suppressed the luciferase activity. Conversely, it did not influence the luciferase activities of those containing mutated seed sequence for miR-1908-5p. Our results reveal that *NKIRAS2* is a novel target gene for miR-1908-5p.

### miR-1908-5p activates the transcriptional activity of NF-κB by suppressing NKIRAS2 expression

**Figure 6:**
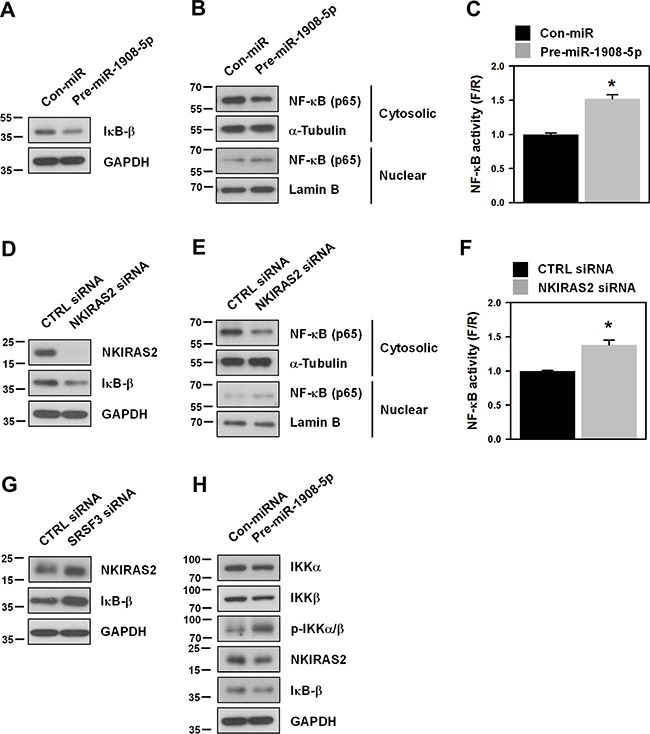
Downregulation of NKIRAS2 by miR-1908-5p is implicated in the oncogenic functions of SRSF3 **A-F**. U2OS cells were transfected with control (CTRL) or SRSF3-specific siRNA (A-C). In the case of miRNA, cells were transfected with control (Con-miR) or pre-miR-1908-5p (D-F). The level of IκB-β was determined by western blot analysis (A and C). To assess the cytoplasmic and nuclear localization of NF-kB, cellular fractionation was performed and the level of NF-κB (p65) in cytoplasmic and nuclear fraction was determined by western blot analysis (B and D). The level of α-tubulin and lamin B was checked for cytoplasmic and nuclear markers, respectively. Transcriptional activity of NF-κB was assessed by checking the luciferase activity as described in Materials and Methods (C and F). **G**. U2OS cells were transfected with control (CTRL) or SRSF3-specific siRNA and the level of NKIRAS2 and IκB-β was determined by western blot analysis. **H**. U2OS cells were transfected with control (Con-miR) or pre-miR-1908-5p. After 48 h post-transfection, IKKα/β activation and expression of NKIRAS2 and IκB-β were assessed by western blot analysis.

Since NKIRAS2 is known to negatively regulate the NF-κB pathway by stabilizing IκB-β and inhibiting nuclear localization of NF-κB (p65), we checked the level of IκB-β and localization of NF-κB (p65) in miR-1908-5p-overexpressed cells. As expected, the expression level of IκB-β was decreased by overexpression of miR-1908-5p (Figure 6A). In accordance with a diminished level of IκB-β, cytoplasmic localization of NF-κB (p65) was decreased (Figure 6B) and the transcriptional activity of NF-κB was increased (Figure 6C). Knockdown of NKIRAS2 similarly decreased the level of IκB-β and cytoplasmic NF-κB (p65), and activated the transcriptional activity of NF-κB (Figures 6D, and 6F, respectively). These results indicate that the miR-1908-5p-mediated suppression of *NKIRAS2* expression induces transactivation of NF-κB by destabilizing IκB-β and inducing nuclear localization of NF-κB (p65). Following transfection with SRSF3 siRNA or pre-miR-1908-5p, the expression level of NKIRAS2 and IκB-β was assessed by western blot analysis. In SRSF3-silenced cells, NKIRAS2 and IκB-β were upregulated as compared to control, which is partly resulted from decreased expression of miR-1908-5p (Figure 6G). Conversely, transfection with miRNA mimic (pre-miR-1908-5p) activated IKKα/β and NF-κB by inducing their phosphorylation. It also suppressed the expression of NKIRAS2 and thus downregulated IκB-β, which indicating that miR-1908-5p is able to activate NF-κB pathway (Figure 6H).

### miR-1908-5p-mediated downregulation of NKIRAS2 is involved in the oncogenic functions of SRSF3

**Figure 7:**
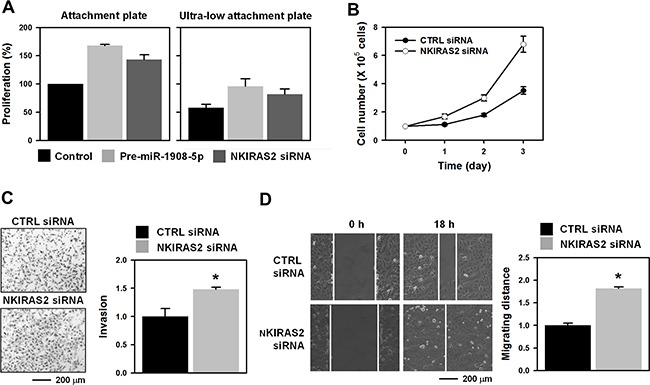
Downregulation of NKIRAS2 by miR-1908-5p is implicated in the oncogenic functions of SRSF3 **A**. To check the effect of miR-1908-5p/NKIRAS2 on the anchorage-independent proliferation, U2OS cells were transfected with pre-miR-1908-5p or NKIRAS2-specific siRNA for 24 h. Equal numbers of transfected cells were plated in attachment or ultra-low attachment plates. After 48 h post-incubation, cellular viability was calculated by the MTS assay. **B**-**D**. U2OS cells were transfected with control (CTRL) or NKIRAS2-specific siRNA for 48 h. Cellular proliferation was calculated by counting viable cells every 24 h (B). Invasive and migratory abilities were assessed by Matrigel invasion (C) and wound healing assays (D), respectively. All experiments are performed more than three times and data represent the mean ± S.D. Asterisk (*) indicates statistical significance of p<0.05 as determined by Student's t-test.

Next, we checked the role of the miR-1908-5p/NKIRAS2/IκB-β axis on the oncogenic functions of SRSF3. As with the knockdown of SRSF3, anchorage-dependent or anchorage–independent cellular proliferation were enhanced by both overexpression of miR-1908-5p and knockdown of NKIRAS2 (Figure 7A and 7B). Invasive and migratory abilities were also potentiated by knockdown of NKIRAS2 (Figures 7C and 7D, respectively). These results suggest that miR-1908-5p-elicited suppression of NKIRAS2 is responsible for the oncogenic function of SRSF3.

**Figure 8:**
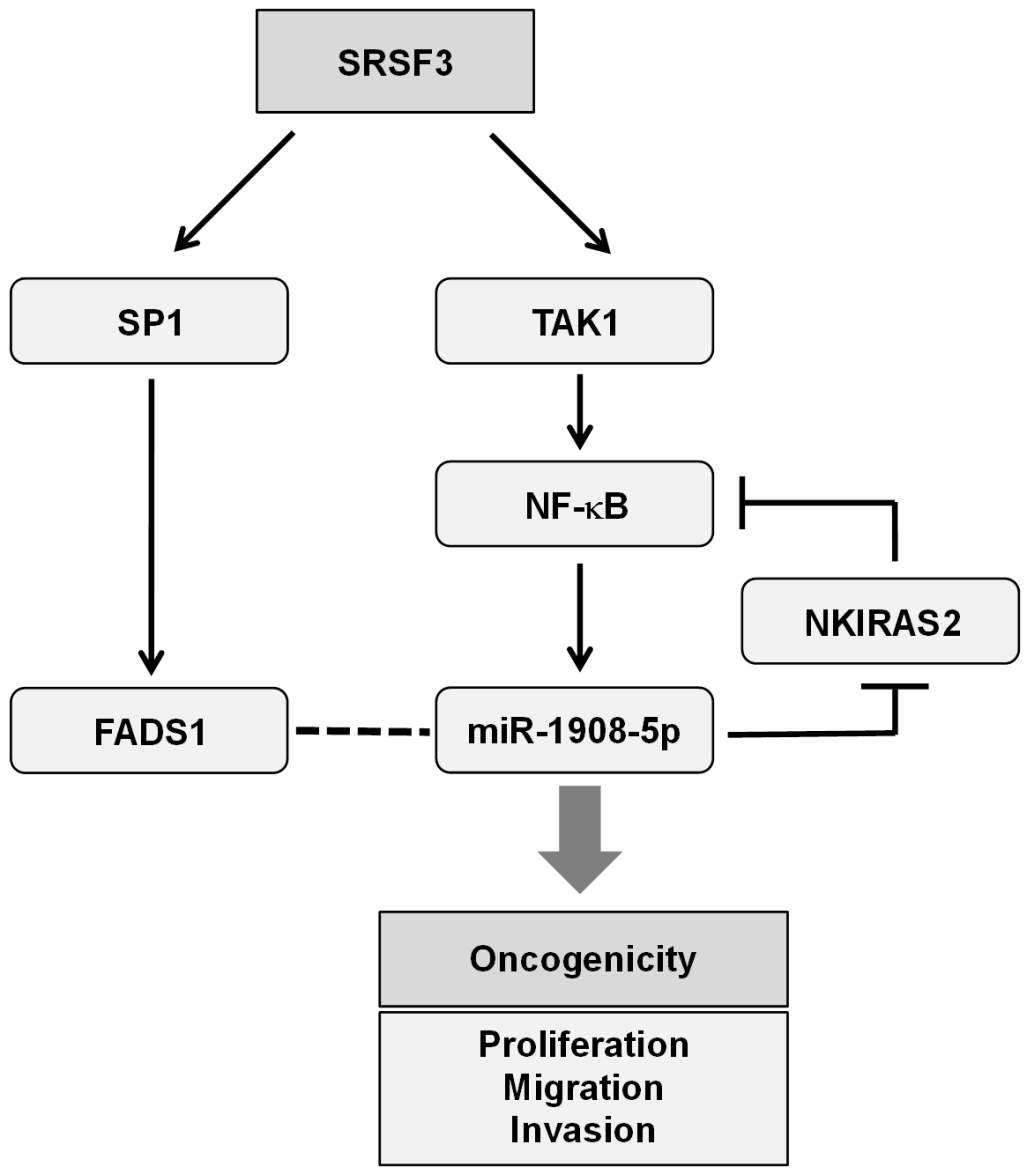
Proposed model for the role of miR-1908-5p in the oncogenic function of SRSF3

From the above results, we concluded that SRSF3 plays a critical role in cancer progression and confers malignant phenotypes on osteosarcoma U2OS cells. It was also found that miR-1908-5p, an NF-κB-dependent oncogenic miRNA, is involved in oncogenic functions of SRSF3 and suppresses NKIRAS2 that potentiates the SRSF3-mediated transactivation of NF-κB. In conclusion, our results suggest that the SRSF3/miR-1908-5p/NKIRAS2 axis is closely associated with the oncogenic functions of SRSF3 and is a promising target for cancer treatment. We proposed the molecular mechanism underlying oncogenic function of SRSF3 through NF-κB/miR-1908-5p/NKIRAS2 axis (illustrated in Figure 8).

## DISCUSSION

Emerging evidence has established that abnormal expression of SR proteins play a critical role in cancer progression mainly by affecting alternative splicing patterns of key transcripts. SR family proteins consist of 12 identified members in human and are characterized with RNA-binding domains and downstream RS domains [2]. The biological relevance of SR proteins is supported by researches proving that aberrant alternative splicing is closely linked with cancer progression. As an oncogene, SRSF3 plays a critical role in cell proliferation by promoting the G2/M transition and preventing apoptotic cell death [4]. In addition to its functions in alternative splicing process, SRSF3 is reported to control the mRNA translation [18] and transcription termination [19]. Several reports demonstrate that SRSF3 is an adapter protein for TAP-dependent export of mRNA [20–22] and is also involved in mRNA adenylation and therefore influences pre-mRNA processing [23].

Through genome-wide analyses of SRSF3 target genes, SRSF3 is reported to significantly influence the expression of a subset of oncogenic or tumor suppressive miRNAs [15]. Given the knowledge that pri-miRNAs are generally transcribed by RNA polymerase II and most miRNAs are located in the intron regions of genes, their expression levels are dependent on splicing events [24]. SRSF3 can regulate the expression of miRNA by controlling transcriptional or splicing events. In addition to its functions at the post-transcriptional level, SRSF3 is also known to influence miRNA biogenesis processes by regulating the Drosha-mediated cleavage of pri-miRNA [25]. In this report, SRSF3 was found to regulate the expression of miR-1908 and its host gene *FADS1*. However, knockdown of FADS1 did not influence the expression of miR-1908, indicating that these two SRSF3 targets are not closely associated. Moreover, *FADS1* was not implicated in the oncogenic functions of SRSF3 either.

Most intronic miRNAs are known to be transcribed together with their host genes [26]. However, the level of several miRNAs are not correlated with the expression of its host gene, indicating that miRNAs could have their own transcriptional factors [27]. By investigating the oncogenic function of SRSF3, we previously observed that SRSF3 regulates a wide range of genes associated with cancer progression [9]. Among them, *FADS1* is a fascinating target gene since it contains the sequence of miR-1908 in its intron. miR-1908 is an intronic miRNA encoded in the intron of *FADS1* and is highly expressed in mature human adipocytes. It was recently reported that two putative binding sites of NF-κB are located in the promoter region of miR-1908 [16]. These findings suggest that miR-1908 is regulated by its own transcription factor, NF-κB. It was also observed that knockdown of SRSF3 inhibited the activation of IKKα and IKKβ and thus diminished the level of phosphorylated NF-κB (p65), which resulted in the cytosolic retention of NF-κB (p65). From these data, we demonstrate that SRSF3 is able to regulate the expression of miR-1908 by activating the NF-κB pathway.

The NF-κB pathway is known to be closely associated with cancer development and progression. By interacting with inhibitors of NF-κB (IκBs such as IκBα, IκBβ, and IκBε), NF-κB dimers are sequestered in the cytoplasm and thus transcriptionally inactive in most types of cells [28]. During the activation of NF-κB signaling, IκBs are phosphorylated by the IκB kinase (IKK) complex (two catalytic subunits, IKKα and IKKβ, and one regulatory subunit, IKKγ) and are degraded through the ubiquitin-proteasome pathway. Upon degradation of IκB, NF-κB dimers translocate into the nucleus where they activate transcription of their target genes that are involved in cancer progression. Aberrant NF-κB activation is tightly associated with malignant conversion of premalignant cells and the production of cytokines, growth factors, proteases, and angiogenic factors in environmental cells [29, 30]. Constitutively active NF-κB is also implicated in the gain of resistance against chemotherapeutics and radiation. NKIRAS2 is known as a potent regulator of the NF-κB pathway by preventing the degradation of IκB, which is responsible for increased protein stability of IκB. In addition to transcriptional activation of its target genes, the NF-κB pathway also plays central roles in cancer progression through upregulation of miRNAs. Abnormal activation of NF-κB is closely implicated in biogenesis of cancer-associated miRNAs such as miR-146a [31, 32], miR-125b [32], miR-155 [33], miR-21 [34], miR-301a [35], miR-9 [36], miR-199a [37], and miR-15/16 cluster [38].

NKIRAS2 is an atypical Ras-like protein that functions as a negative regulator of the NF-κB pathway by hindering the phosphorylation-dependent degradation of NF-κB inhibitor beta (NFKBIB, also termed IκB-β). Therefore, NKIRAS2 inhibits the transactivation of NF-κB by stabilizing IκB-β and thus preventing the nuclear translocation of NF-κB (p65). We observed here that miR-1908-5p suppressed the expression of NKIRAS2 through direct binding to the 3’UTR of its mRNA, which indicates that NKIRAS2 is a novel target of miR-1908-5p. Taken together, SRSF3 confers malignant phenotypes on cancer cells by upregulating the NF-κB-dependent miR-1908-5p expression and its oncogenic functions are enforced by positive feedback regulation of the NF-κB pathway via the SRSF3/miR-1908-5p/NKIRAS2 axis.

## MATERIALS AND METHODS

### Cell culture and transfection

Human osteosarcoma U2OS cells were purchased from Korean Cell Line Bank (Republic of Korea) and maintained in Dulbecco's modified Eagles medium (high glucose, Hyclone, GE Healthcare, Little Chalfont, UK) supplemented with 10% fetal bovine serum and 1% antibiotic-antimycotic solution (Gibco-BRL, Thermo Fisher Scientific, Waltham, MA, USA). U2OS cells were authenticated through short tandem repeat profiling.

For siRNA transfection, U2OS cells were plated at adequate density and transfected with the indicated siRNAs or control (CTRL) siRNA using Lipofectamine2000 (Invitrogen, Thermo Scientific) according to the manufacturer's protocol. The siRNAs for SRSF3 (GAGUGGAACUGUCGAAUGG) and NKIRAS2 (CGUCCUGGUCUAUAGCACA) were synthesized by Bioneer (Daejeon, Republic of Korea). FADS1-targeting (sc-96474) and Sp1-targeting siRNA (sc-29487) were purchased from Santa Cruz Biotechnology (Santa Cruz, CA, USA). For overexpression of miRNA, miRNA-1908-5p mimic was purchased from GenePharma (Shanghai, China) and introduced into cells using Lipofectamine2000 (Invitrogen). Transfection of miRNA mimic sufficiently increased the level of miR-1908-5p (more than 1,000-fold increase).

### Cell proliferation and clonogenicity

Transfected cells were resuspended into 12-well plates and the proliferation rate was determined by counting viable cells at every 24 h. For the colony forming assay, an equal number of transfected cells (300 viable cells) were plated in 6-well plates and maintained in medium supplemented with serum. Two weeks later, the colonies were fixed with 4% paraformaldehyde and stained with crystal violet solution. Clonogenicity was assessed by counting the numbers of colonies.

### Determination of metastatic potential

Invasiveness was assessed using a BD Biocoat™ Matrigel® invasion chamber (BD Bioscience, San Jose, CA, USA). Equal cell numbers of transfected cells (5 × 10^4^ cells) were inoculated into the upper well and invasion was initiated by adding serum-containing medium into the lower well. After incubation for 24 h, invading cells were stained with hematoxylin and eosin and photographed under a microscope. Invasive activities were determined by counting the number of invaded cells. For determination of migratory abilities, a wound healing assay was performed. Transfected cells were seeded into 12-well plates with high density and then a straight scratch was produced using a white pipette tip. After incubation for 16 h, the migrated distance was calculated by AxioVision microscope software (ZEISS, Jena, Germany).

### RNA isolation and RT-qPCR

After 48 h post-transfection, total RNA was isolated with TRIzol reagent (Ambion, USA) and was reverse transcribed using SuperScript III First-Strand Synthesis System (Invitrogen, USA). The product was used to perform real-time quantitative-PCR (RT-qPCR) amplification using the specific primers as follows: SRSF3 (forward, 5’-GCATCGTGATTCCTGTCCAT-3’; reverse, 5’-CGGAGTGGTCCATAGTAGCC-3’); FADS1 (forward, 5’-GCTACTTCACCTGGGACGAG-3’; reverse, 5’-GGTGAACTCGCTGATGTTGT-3’); pri-miR-1908 (forward, 5’-CAGACGAAACAGGCACCAAC-3’; reverse, 5’-CACATACGGACCAATCGCCG-3’); Sp1 (forward, 5’-GTGGAGGCAACATCATTGCTG-3’; reverse, 5’-CCACTGGTACATTGGTCACAT-3’); NKIRAS2 (forward, 5’-GAGAGCAGGTGCGTTTCTATG-3’; reverse, 5’-ACCAGGACGTAGCCATCAGT-3’); Cyclin D1 (forward, 5’-CCGTCCATGCGGAAGATC-3’; reverse, 5’-ATGGCCAGCGGGAAGAC-3’); Slug (forward, 5’-TGTTGCAGTGAGGGCAAGAA-3’; reverse, 5’-GACCCTGGTTGCTTCAAGGA-3’); HIF-1α (forward, 5’-CACTACCACTGCCACCACTG-3’; reverse, 5’-TGGGTAGGAGATGGAGATGC-3’); and GAPDH (forward, 5’-TGCACCACCAACTGCTTAGC-3’; reverse, 5’-GGCATGGACTGTGGTCATGAG-3’). For detection of miRNA, TaqMan^®^ gene expression assays were performed using hsa-1908-5p-specific and has-miR-1908-3p specific primers (Applied Biosystems, USA).

### Preparation of cytoplasmic and nuclear extracts

For isolation of cytosolic and nuclear fractions, cells were harvested and lysed with digitonin-containing RSB buffer. After centrifugation at 2,000 × g for 8 min, the supernatant was transferred to a new tube (cytosolic extracts). The remaining pellet was washed four times with RSB buffer and lysed with RIAP buffer. Nuclear extracts (NE) were isolated by centrifugation at 13,200 rpm for 30 min [39].

### Western blot analysis

Whole cell lysates were prepared using RIPA buffer containing phosphatase inhibitor and protease inhibitor cocktail (Roche, Basel, Switzerland). Following standard western blotting procedure, proteins were blotted onto PVDF membrane (Millipore, Bilerica, MA, USA) and recognized by the following antibodies: SRSF3 (sc-13510), α-tubulin (sc-69969), and lamin B (sc-6216) were from Santa Cruz Biotechnology; Sp1 (#5931), IKKα (#2682), IKKβ (#2370); p-IKKα/β (#2697), NF-κB p65 (#8242), p-NF-κB p65 (#3033), Cyclin D1 (#2926), and Slug (#9585) were from Cell Signaling Technology (Danvers, MA, USA); IκB-β (A301-828A) was from Bethyl Laboratories (Montgomery, TX, USA); HIF1-α (610958) was from BD Bioscience; NKIRAS2 (ab57303), GAPDH (ab8245), and β-actin (ab8226) were from Abcam (Cambridge, UK).

### Luciferase reporter assay

To assess the transactivation of NF-κB, a pGL4.32 [luc2P/NF-κB-RE/Hygro] vector (E849A; Promega, Madison, WI, USA) containing an NF-κB responsive element was used. U2OS cells were transfected with siRNA or miRNA for 24 h and then the firefly reporter vectors were transfected along with renilla expressing vector (E2231; Promega).

For validation of miRNA recognition elements, the luciferase constructs containing the binding sites of miR-1908-5p were manufactured using a pmirGLO vector (E133A; Promega). Since two positions are predicted for the miR-1908-5p binding site in the 3’UTR of *NKIRAS2* mRNA, we prepared two individual vectors containing wild-type or mutant sequences of miR-1908-5p. In order to check the effect of miR-1908-5p on NKIRAS2 expression, U2OS cells were seeded in 24-well plates with around 50% confluency and transfected with the indicated siRNA or miRNA. At 24 h post-transfection, cells were transfected with either a pmirGLO blank vector or pmirGLO-NKIRAS2/3’UTR vector (willd-type or mutant). The luciferase activity was measured 24 h later using Dual-Glo Luciferase Assay System (E2940; Promega) on a GloMax^®^20/20 Luminometer (Promega).

### Ago2 immunoprecipitation

To check the interaction between miRNA and *NKIRAS2* mRNA, immunoprecipitation (IP) with Ago2 was performed. Briefly, Ago2 antibody (Sigma-Aldrich, St. Louis, MO, USA) and Dynabeads Protein G (Invitrogen) were incubated at 4°C with rotation a day prior to the experiment. Cytoplasmic lysates of transfected cells were prepared with PEB buffer and incubated with Ago2 antibody-coated beads for 3 h. RNAs in the Ago2 IP materials were isolated and then the level of *NKIRAS2* mRNA was determined by RT-qPCR [39].

## SUPPLEMENTARY MATERIALS FIGURES AND TABLES


