# Supplementary material for: MicroRNA-1908-5p contributes to the oncogenic function of the splicing factor SRSF3
Source: Oncotarget. 2016 Dec 26;8(5):8342–55. doi: 10.18632/oncotarget.14184 (PMC5352405; doi:10.18632/oncotarget.14184)
Supplement: Supplementary file 1 [file oncotarget-08-8342-s001.pdf]

MicroRNA-1908-5p contributes to the oncogenic function of the splicing factor SRSF3

SUPPLEMENTARY FIGURES

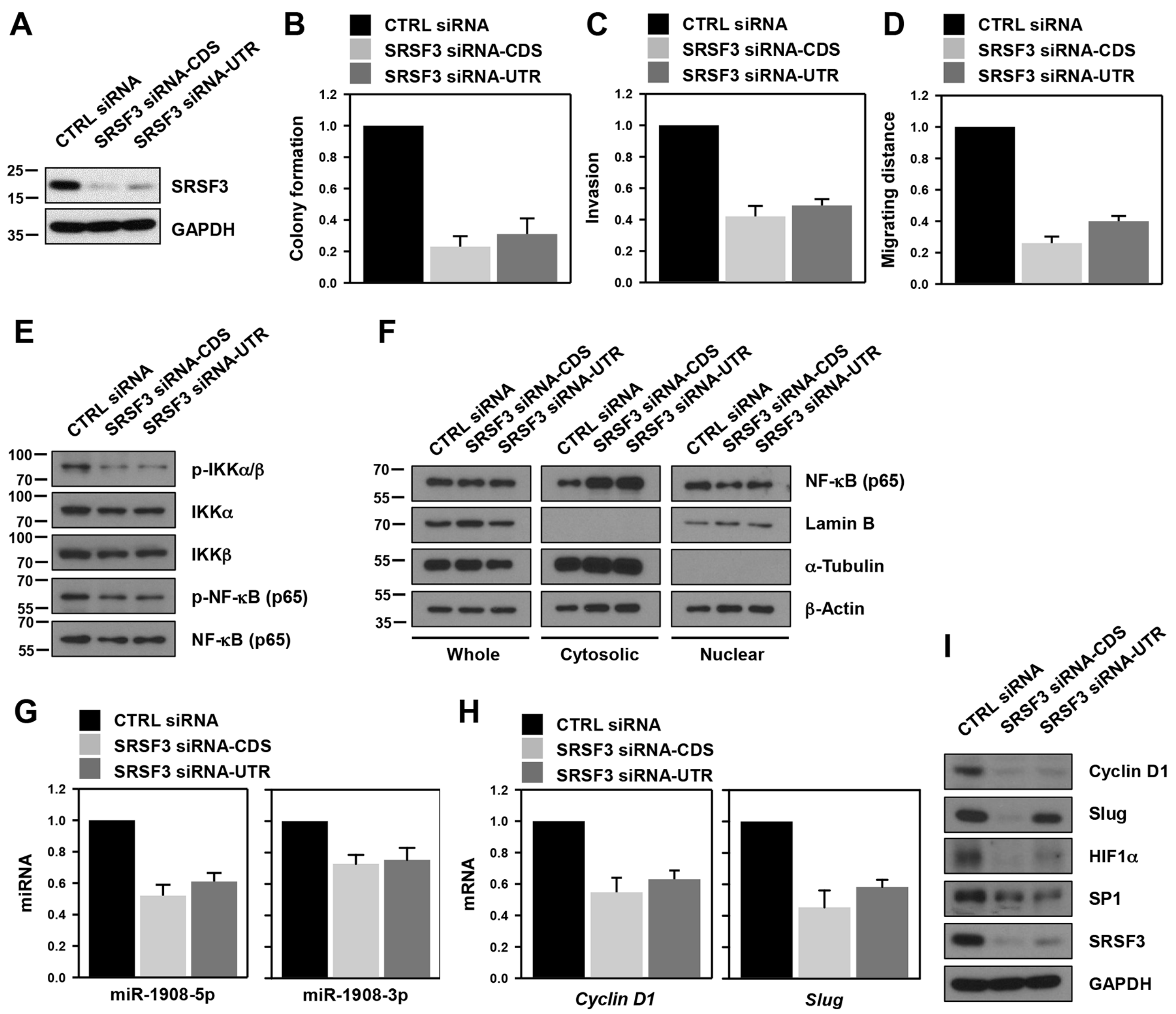

Supplementary Figure 1: Knockdown of SRSF3 by two different siRNA targeting coding region or 3'UTR. Following transfection of U2OS cells with control (CTRL) or SRSF3-specific siRNAs targeting coding sequence (CDS) or 3' untranslated region (UTR), the effect of SRSF3 silencing was investigated: expression level of SRSF3 **A.** clonogenicity **B.** invasive **C.** and migratory **D.** abilities; NF-kB activation **E.** and localization **F.**; miRNA expression **G.** and NF-kB target gene expression (**H.** RT-qPCR; **I.**, western blot).

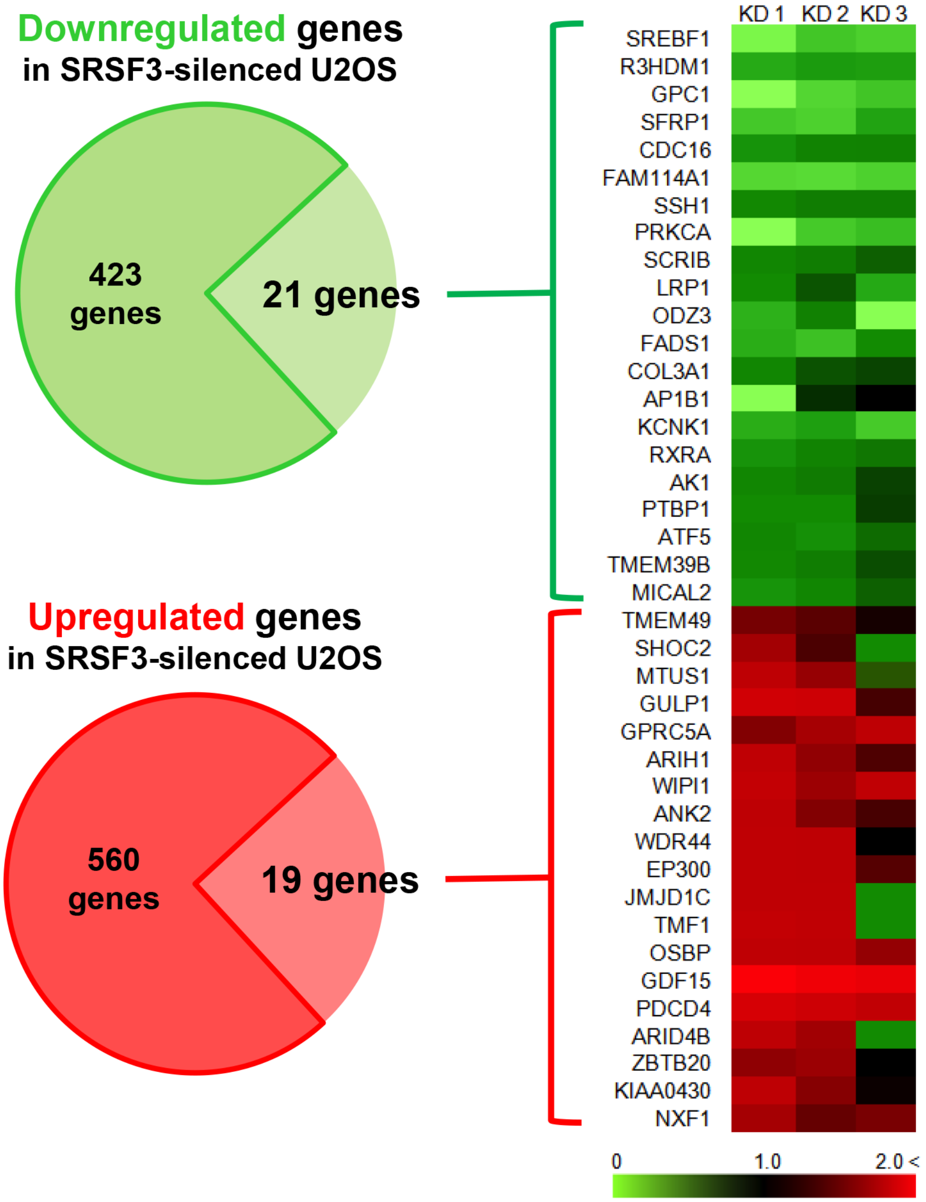

Supplementary Figure 2: List of differentially expressed genes harboring primary miRNA sequences in their intron. It was previously reported the list of differentially expressed genes (DEGs) by knockdown of SRSF3 in U2OS cells. Among them, we screened genes harboring primary sequences of miRNAs and thus identified 21 or 19 genes in 444 downregulated and 579 upregulated genes, respectively.

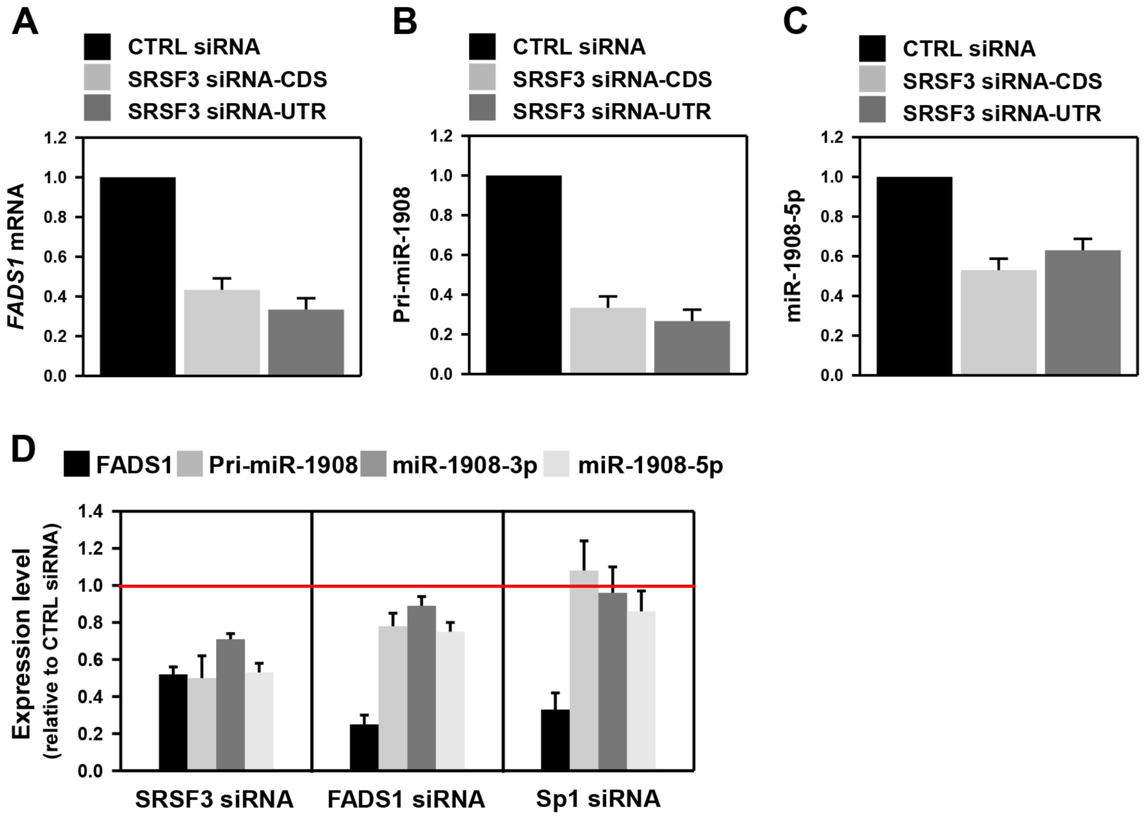

Supplementary Figure 3: SRSF3 regulates the expression of miR-1908 independent of its host gene, FADS1. A-D. U2OS cells were transfected with control (CTRL) or SRSF3-specific siRNA for 48 h. Total RNA was isolated and then the expression level of FADS1 (A) and pri-miR-1908 (B) was determined by RT-qPCR using specific primers. In case of mature miRNA (miR-1908-3p/5p), TaqMan miRNA assay was performed using target specific stem-loop primers (C). The level of GAPDH mRNA and U6 snRNA was used for normalization, respectively. All experiments are performed more than three times and data represent mean ± S.D.

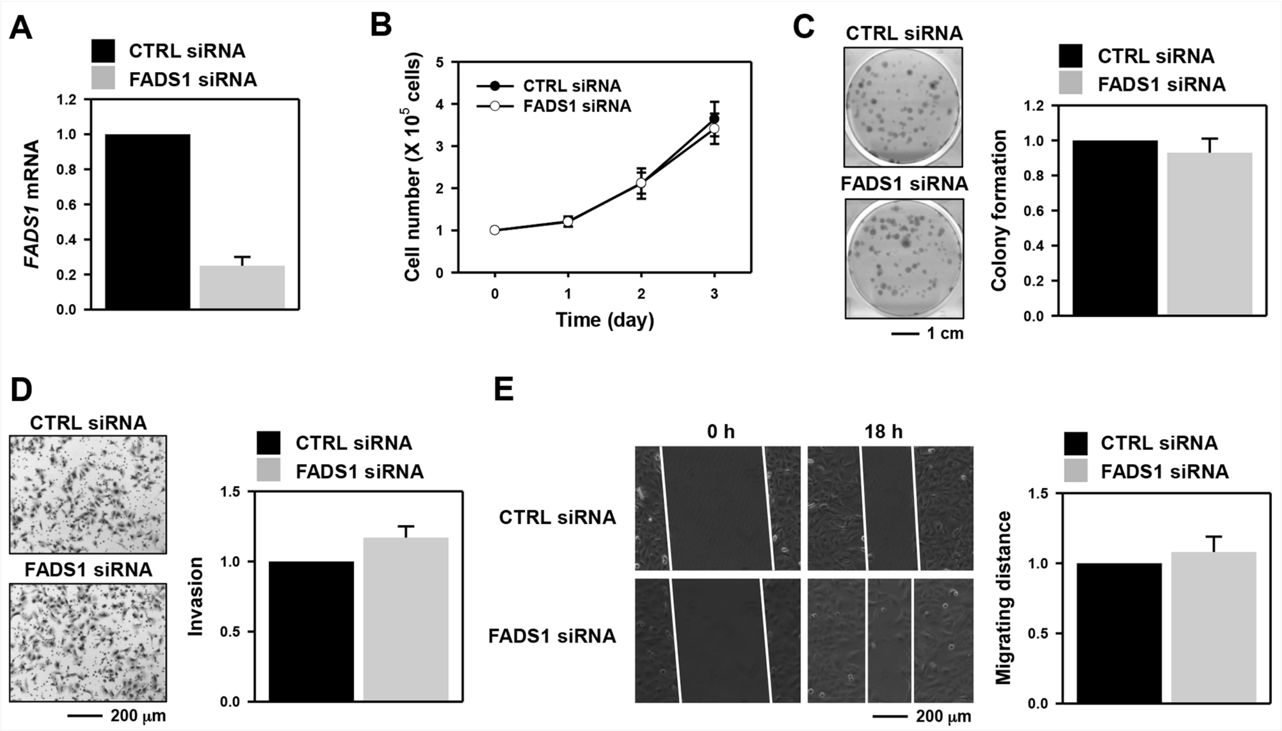

Supplementary Figure 4: FADS1 is not involved in SRSF3-mediated malignant phenotypes of U2OS cells. A. U2OS cells were transfected with control (CTRL) or FADS1-specific siRNA. The expression level of SRSF3 was determined by RT-qPCR. **B.** Equal number of transfected cells was resuspended into 12-well plates and cellular proliferation was assessed by counting the number of viable cells at every 24 h. **C.** For clonogenic assay, three hundreds of transfected cells were plated into 6-well plates and cultured for more than 2 weeks. Clonogenic activity was assessed by counting the number of colonies. **D-E.** The invasive (D) and migratory (E) abilities were assessed by matrigel invasion assay and wound closure assay, respectively, as described in Materials and Methods. All experiments are performed more than three times and data represent mean ± S.D.
